# Anti-biofouling NH_3_ gas sensor based on reentrant thorny ZnO/graphene hybrid nanowalls

**DOI:** 10.1038/s41378-020-0151-5

**Published:** 2020-07-13

**Authors:** Tian Hang, Jiangming Wu, Shuai Xiao, Baohong Li, Hongbo Li, Chengduan Yang, Cheng Yang, Ning Hu, Yonghang Xu, Yu Zhang, Xi Xie

**Affiliations:** 1 State Key Laboratory of Optoelectronic Materials and Technologies, School of Electronics and Information Technology, Guangdong Province Key Laboratory of Display Material and Technology, Sun Yat-Sen University, Guangzhou, 510006 China; 2 School of Materials Science and Energy Engineering, Foshan University, Foshan, 528000 China; 3 The First Affiliated Hospital of Sun Yat-Sen University, Guangzhou, 510080 China

**Keywords:** Electronic properties and materials, Electronic properties and materials

## Abstract

Since toxic gas leakage may cause ecological environmental problems and even life-threatening damage, effective monitoring of toxic gas is of great importance and subject to increasing demand. However, complicated environmental factors, as well as various coexisting interferences can easily affect the sensitivity and selectivity of gas sensors, hindering their performance. Recent reports have successfully demonstrated the development of hierarchical nanostructures with desirable self-cleaning properties, yet gas sensors that can resist contamination have rarely been realized. Here, we developed a reentrant thorny ZnO/graphene hybrid nanowall structure that simultaneously repels liquid contamination and possesses NH_3_ gas sensing properties. The unique reentrant and hierarchical structure, featuring an interconnected vertical graphene nanowall framework with numerous ZnO nanospikes branched on the top nanowall, is highly repellent to liquids, even biofluids with low surface tension. The hierarchical structure consisting of gas sensing graphene and ZnO can be successfully applied as an NH_3_ gas sensor at room temperature, exhibiting not only excellent sensitivity, selectivity, and repeatability, but also outstanding stability even after bacterial contamination. This study provides a versatile method for fabricating reentrant and hierarchical structures with excellent liquid repellency, and offers a promising method for designing reliable gas sensors with anti-biofouling properties.

## Gas sensors: a self-cleaning pollution monitor

A hybrid nanostructure can simultaneously repel contaminants and possess ammonia-sensing capabilities, making it an ideal pollution monitor. Gaseous pollutants such as ammonia have significant medical and environmental impacts. Hybrid structures of metal oxides and graphene have shown promising gas-sensing performance; however, their utility is hampered by the complexity of air composition and the presence of other contaminants. Xi Xie, of Sun Yat-Sen University, China, led a team of researchers to create a nanostructure of zinc oxide and graphene — both known as ammonia gas-sensitive materials — that repels liquids from its surface. The structure works by trapping “nano-air pockets” on the face of the device that prevent liquids from adhering to it, while the presence or absence of ammonia changes the conductivity of the nanostructure. The researchers’ paper shows an effective sensor for use in complicated environments.

## Introduction

Toxic gases, such as ammonia (NH_3_), originating from exhaust gases in a variety of farming and industrial processes, including pharmaceutical and fertilizer manufacturing, can cause ecological environmental problems and life-threatening conditions^1,2^. For example, exposure to an NH_3_ concentration of 25 ppm can lead to skin, eye, and lung irritation in the human body^3^. Prolonged exposure to high concentrations of toxic gases can even endanger human life, and thus, it is of great importance to monitor their concentrations. The emergence of gas sensors has enabled the conversion of chemical information about a specified gas into analytical signals so that the concentrations of toxic gases can be accurately detected and evaluated. If excessive amounts of toxic gases are present, an alarm associated with the gas sensor can be triggered. Recently, graphene has been demonstrated to have unique properties, including high charge-carrier mobility, a large surface area, and high thermal stability, making it a highly promising material for gas sensing applications, especially for detecting nitrogen-based gases^2,4–7^. However, certain limitations, such as long recovery time and irreversibility, have hindered the further application of graphene in robust gas sensors^8^. Metal oxides (MOs), such as ZnO, SnO, and WO, are also widely studied and utilized as gas sensing materials for toxic gas monitoring because of their advantages, including low cost and versatile design^3,9–13^. To date, many MO gas sensors have been introduced and have greatly promoted the progression of gas sensing technology, yet these sensors are still limited by their unsatisfactory sensitivity and selectivity, inherent high resistance, and relatively high working temperature^14^. To compensate for the deficiency of a single material alone, gas sensors based on hybrid materials developed by the integration of graphene and MO semiconductors have been proposed^14–17^. For example, enhanced gas sensing properties of SnO_2_/graphene composites at room temperature (RT) have been reported^18^. High-performance NH_3_ and NO_2_ sensors based on ZnO nanostructures, and reduced graphene oxide (rGO) composites have also each been demonstrated^15,16^.

Despite this progress, accurate detection of toxic gases still presents challenges due to the complicated air composition in real environments, and potential fouling or damage to sensors by the environment. For example, in poultry farms, NH_3_ emissions can be highly concentrated due to the confined animal production in small and limited geographical areas^19^. Accurate estimates of NH_3_ emissions are essential but difficult to obtain under commercial poultry house conditions. Coexisting gases, as well as interferents, including water vapor, solid particles, dust, and microorganisms in the air, can significantly affect the detection accuracy and performance of gas sensors. Once these undesired interferents are adsorbed or attached on the active material of the sensing device, the underlying sensing functionality of the gas sensor can be inhibited^20^. Merely increasing the surface roughness or specific surface area is not sufficient for building a stable and persistent sensing interface, although such changes may improve the sensitivity^21^. To date, some anti-biofouling biosensors with biocompatible or self-cleaning chemical coatings have been reported to provide improved sensing in liquid environments without being contaminated; however, the development of reliable gas sensors with resistance to contaminants in air or gas environments is still highly challenging, yet in great demand. In addition, superhydrophobic chemical coatings used for self-cleaning can generally passivate the sensing materials, presenting a barrier that may reduce the direct access of target molecules to the sensing materials, and thus potentially compromise the sensitivity or specificity of the sensor. The development of anti-biofouling sensors without additional chemical coatings has rarely been achieved.

Topographical features play an important role in the interaction between gases and surfaces^15,22^. Special geometries, such as hierarchical and reentrant structures, have shown favorable antifouling properties^23–25^. Such structures can form a composite solid–liquid–air interface that can effectively prevent liquids from penetrating and reduce the nonspecific adsorption of interfering substances^23,26^. Although superhydrophobic or superomniphobic chemical coatings can effectively prevent the adhesion of liquids or contaminants, passivation of the sensor surface by the chemical coating would block the sensor and interfere with sensing functionalities^27^. On the other hand, unlike traditional chemical modification approaches that require low-surface-energy reagents (e.g., fluorine-containing coatings), reentrant structures have been reported to possess excellent antifouling properties even without low-surface-energy coatings^24,28^. Antifouling reentrant structures are promising candidates for applications in designing functional sensors because the surface chemical properties will not undergo subsequent chemical modification, resulting in minimal changes in sensing functionalities.

**Fig. 1:**
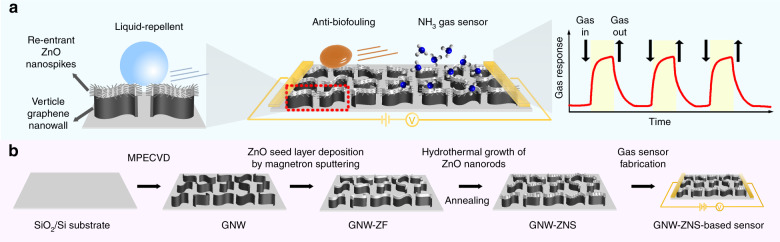
Schematic illustration and fabrication process of the GNW-ZNS-based sensor. **a** Schematic illustrations of an antifouling gas sensor based on the hierarchical reentrant GNW-ZNS structure. **b** The fabrication process of the GNW-ZNS-based sensor: vertical GNWs were prepared on a Si/SiO_2_ substrate, followed by ZnO seed layer deposition using magnetron sputtering, hydrothermal growth of ZnO nanospikes, and annealing. GNW-ZNS was further integrated and used as a gas sensor for NH_3_ detection

In this article, we have developed a reentrant thorny ZnO/graphene hybrid nanowall structure (denoted as GNW-ZNS) and demonstrated its excellent liquid-repellent and NH_3_ gas sensing properties. The thorny nanowall structure consists of a vertical graphene nanowall (GNW) framework with numerous ZnO nanospikes (denoted as ZNS) branched on top of the nanowalls (Fig. 1a). Because of its unique reentrant and hierarchical characteristics, GNW-ZNS exhibits excellent liquid repellency toward various fluids without additional surface modification. By combining the advantages of gas sensing graphene and ZnO, GNW-ZNS can be successfully applied as an NH_3_ gas sensor at RT with reasonable sensitivity, and excellent selectivity and repeatability. Moreover, owing to the outstanding liquid repellency from the reentrant structure, the GNW-ZNS-based sensor also displays high stability with a good response even after severe bacterial contamination. This study offers a facile method for fabricating hierarchical and reentrant structures with robust hydrophobicity and liquid repellency and provides a unique strategy to engineer advanced gas sensors with reliable performance for extended applications.

## Results and discussion

**Fig. 2:**
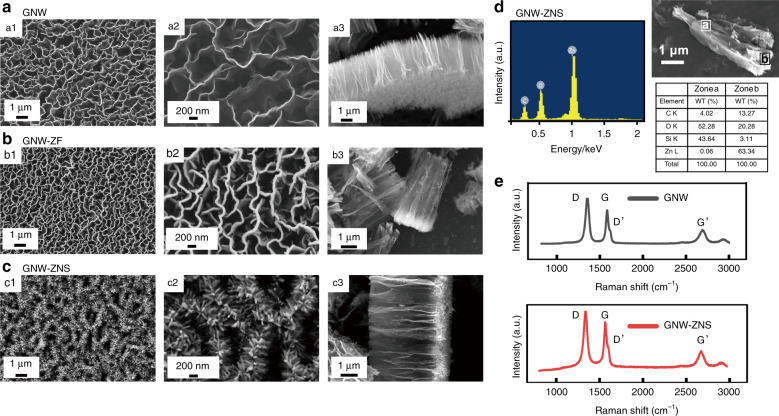
Morphology and composition characterization of GNW-ZNS. **a**–**c** SEM images of GNW, GNW-ZF, and GNW-ZNS: (a1–2) top views and (a3) side view of GNW showing the vertical nanowall structure; (b1–2) top views and (b3) side view of GNW-ZF; the ZnO layer was deposited on the top of the GNWs; (c1–2) top views and (c3) side view of GNW-ZNS; ZnO spikes were grown on the top portion of the GNW. The reentrant features can be observed in c3. **d** EDS spectroscopy of GNW-ZNS (substrate: Si/SiO_2_) confirmed the chemical composition of Zn, O, and C, showing that ZnO could only form on the top part. **e** Raman spectra confirmed the few-layer graphene structures in both GNW and GNW-ZNS

The hierarchical and reentrant GNW-ZNS was fabricated in three main steps (Fig. 1b), and the morphology of the obtained structures was characterized by scanning electron microscopy (SEM; Fig. 2a–c). Vertically aligned GNWs were first grown on SiO_2_/Si substrates by the microwave plasma enhanced chemical vapor deposition (MPECVD) method. As shown in Fig. 2a, nanowalls with a height of ~3.2 µm and edge length of ~1.3 µm uniformly stood, and were distributed on the SiO_2_/Si substrates. These interwoven nanowall sheets connected to each other, forming open and porous networks with an average pore size of ~780 nm. In the second step, a thin film of ZnO was deposited on the GNWs by magnetron sputtering (sample denoted as GNW-ZF). Due to the steric effect of the vertical nanowall structure with narrow spacing between the nanowalls, this ZnO layer could neither penetrate the bottom surface nor adhere to the sidewall surface of the nanowalls. Instead, only the top portion of the nanowalls was covered with ZnO (Fig. 2b). This ZnO layer served as the nucleation/seed layer for the subsequent growth of ZnO nanorods by a hydrothermal reaction in an aqueous solution of Zn(NO_3_)_2_ and diethylenetriamine. After the reaction, a reentrant thorny ZnO/graphene hybrid nanowall structure was obtained, whose surface morphology after annealing at 150 °C is shown in Fig. 2c. In the final structure, numerous ZnO nanothorns with an average length of 220 nm and diameter of 60 nm were grown on top of the nanowalls. The resulting thorny nanowall-like hierarchical structure not only retained the characteristic structure of the vertical nanowalls from graphene, but also possessed an increased surface area and reentrant features because of the ZnO nanothorns introduced on the graphene top edge.

The chemical composition was investigated by energy dispersive X-ray spectroscopy (EDS), confirming the existence of C, Zn, and O and showing that ZnO could only form on the top part of the GNW-ZNS (Fig. 2d). In addition, Raman spectra were recorded to further characterize the graphene structure in the GNW and GNW-ZNS (Fig. 2e). For the GNW, the characteristic peaks of graphene, including the G band located at ~1578 cm^−1^ and the Gʹ band (or 2D band) located at 2687 cm^−1^, were observed, demonstrating its few-layer graphene structure with an I(Gʹ)/I(G) intensity ratio of 0.43 (ref. ^29^). The defect-related D band peak at 1347 cm^−1^ and the small Dʹ band peak at 1584 cm^−1^ indicated the presence of abundant defects, possibly from the graphene edges^30,31^. The Raman spectrum of GNW-ZNS was similar to that of GNW, suggesting that the graphene structure in GNW-ZNS remained intact without significant changes. In addition, because of the high controllability of the MPECVD, magnetron sputtering, and hydrothermal growth processes, the morphology of the obtained GNW-ZNS could also be controlled. For example, GNW-ZNS samples with different spacings could be obtained (Supplementary Fig. 1, Supplementary Table 1). In this study, GNW-ZNS with a moderate inter-nanowall spacing was deliberately chosen for subsequent study. This selection was made because an open and porous vertical nanowall structure with high surface area and sufficient spacing may provide favorable conditions for both antifouling and gas sensing.

**Fig. 3:**
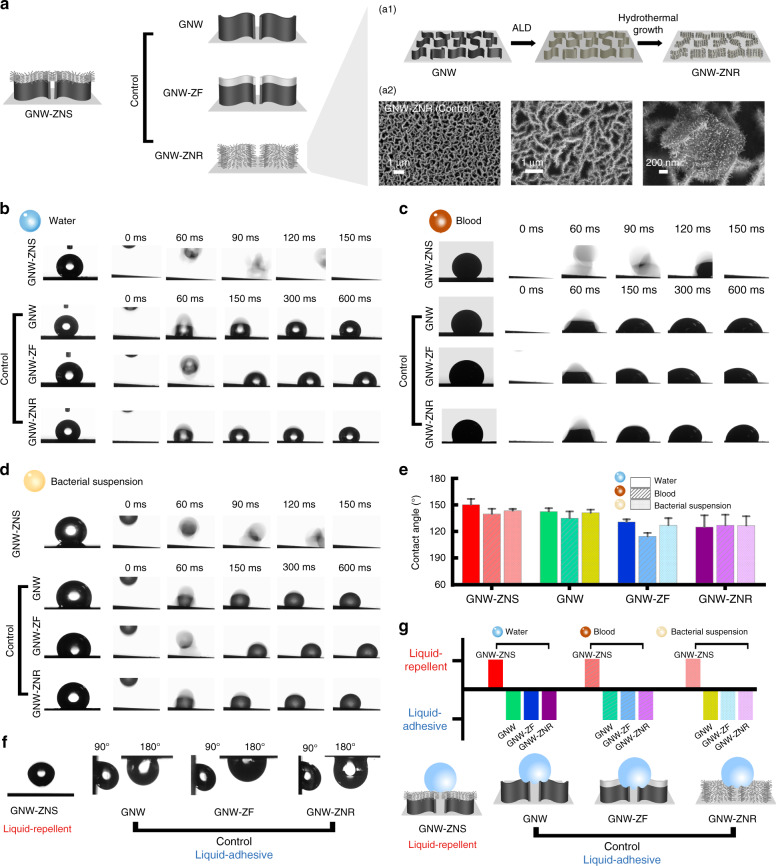
Wettability studies of GNW-ZNS. **a** Different structures tested in the wetting tests: GNW-ZNS and the control samples GNW, GNW-ZF, and GNW-ZNR. a1 GNW-ZNR was fabricated by ALD and hydrothermal growth. a2 SEM images indicated that the GNW-ZNR ZnO nanorods were uniformly distributed on the graphene nanowalls. **b**–**d** Optical images of the static contact angle (left) and time-resolved images of droplets impacting surfaces at a tilted angle of 2° (right) using **b** water (5 μl), **c** blood (12 μl), and **d** a bacterial suspension (5 μl). **e** Statistical results of the CAs. **f** Optical images showing that a water droplet (5 μl) could jump off GNW-ZNS but was suspended on the control GNW, GNW-ZF, and GNW-ZNR surfaces, even at tilt angles of 90° and 180°. **g** Summary of wetting properties of different structures. All liquid drops could quickly bounce off GNW-ZNS but were stuck on the control GNW, GNW-ZF, and GNW-ZNR surfaces. Schematic showing the proposed wetting states on different structures

The surface morphology and properties can determine wetting behaviors, and can influence gas sensing performance^22,32^. The wettability of the different surfaces was systematically studied. The contact angle (CA) with various liquids, including water, a bacterial suspension and blood, was measured for GNW-ZNS and compared with that of control samples, including GNW and GNW-ZF (Fig. 3a). To investigate the role of the reentrant feature in wettability, GNWs without overhanging suspended nanospikes but with uniformly distributed ZnO nanorods (denoted as GNW-ZNR) were fabricated and compared with GNW-ZNS. Briefly, GNW-ZNR was fabricated by multiple steps, including MPECVD of GNW, atomic layer deposition (ALD) of a ZnO seed layer, and ZnO nanorod branching by hydrothermal growth. The CA was determined using a Kruss DSA goniometer by placing a 4 µl droplet onto the sample surface in air at RT (Fig. 3a)^31^. To show the difference in the liquid-to-solid adhesion characteristics of different samples, optical photos showing liquid droplets released from a height of 6 cm and impacting tilted surfaces (tilted angle = 2°) are also presented.

As shown in Fig. 3b–d, among all samples, GNW-ZNS displayed the highest hydrophobicity, with the highest water CA of 150.4 ± 6.6°. The control samples were less hydrophobic, with water CAs of 142.6 ± 3.9° for GNW, 130.9 ± 3.0° for GNW-ZF, and 125.2 ± 13.3° for GNW-ZNR. In addition, relatively high CAs (>139°) were obtained on GNW-ZNS for low-surface-tension liquids, including blood (surface tension = 51.6 mN m^−1^ at 298 K) and a bacterial suspension (surface tension = 66.3 mN m^−1^ at 298 K). Although all samples displayed hydrophobic properties, with CAs larger than 90°, the wetting behaviors of the surfaces differed considerably from those of GNW-ZNS and the other samples (Supplementary Table 2). The GNW-ZNS surface showed high liquid repellency, and all tested liquid drops quickly and readily bounced off the GNW-ZNS surface. In contrast, on the control samples, the liquids could not roll off and tended to become stuck. The control GNW, GNW-ZF, and GNW-ZNR surfaces were highly adhesive to the liquid drops. As shown in Fig. 3f, a 5 µl water droplet remained firmly pinned even when the surfaces were tilted at 90° or turned upside down. Our results showed that GNW-ZNS was highly anti-wetting, while the control samples exhibited a relatively high CA and high adhesion. In nature, similar wetting phenomena have been found, i.e., the “lotus effect” and the “rose petal effect”^33^. Lotus leaves are superhydrophobic, and water droplets can easily roll away with an ultra-low sliding angle. Rose petals can support a water droplet with a large CA and high liquid affinity, and even if the petals are turned upside down, water droplets can remain on the surfaces. In this study, GNW-ZNS showed the “lotus effect”, and the other control samples were similar to rose petals. The possible reason for this result is discussed as follows.

Graphene is commonly considered a hydrophobic material, and the water CA on graphene can be as high as ~127° (refs. ^34,35^). The GNW material initially obtained by MPECVD displayed a relatively high water CA of 142.6°, possibly because the nano-gaps or nano-voids between the vertical nanowalls in GNW could trap air, leading to high CAs. Nevertheless, many of these pores in GNW were on the micrometer scale, and liquid may still penetrate these micro-gaps, causing strong adhesion. This wetting behavior of GNW was very similar to that of natural rose petals with micro-papillae, where liquid can impregnate larger grooves between micro-papillae but cannot enter smaller grooves. Our observations were also consistent with a previous report that flower-like few-layer graphene nanoclusters on nanocone arrays show a high CA and ultrahigh adhesion^36^. Wettability is determined not only by morphological characteristics but also by material properties. Since the ZnO seed layer in GNW-ZF was relatively thin (~30 nm) compared with that of GNW, the local roughness of GNW-ZF was altered slightly; therefore, the change in CA was mainly caused by the ZnO. The surface free energy of ZnO is much higher than that of graphene^34,37^. Therefore, water may penetrate into the ZnO nanowall edge more deeply in GNW-ZF than in GNW, resulting in a decrease in CA, although ZnO was only deposited on the top fraction of the GNW. After hydrothermal growth of ZNS and moderate annealing, the hierarchical GNW-ZNS sample achieved excellent hydrophobicity with high CAs. Moreover, in contrast to GNW and GNW-ZF, the GNW-ZNS surface was highly liquid repellent (Fig. 3f), indicating that the hierarchical reentrant structure helped the hydrophobicity. Through hydrothermal growth, a large number of ZNS were grown on top, and accordingly, numerous small air pockets could form. Compared with GNW and GNW-ZF, the inter-nanowall spacing in GNW-ZNS decreased, and the initially exposed micro-sized pores or voids were trapped or blocked at the bottom due to the nanothorns introduced above. Therefore, the underlying nano- and micro-gaps could lock air more efficiently. In other words, the nano-air pockets created by the nanothorns on the upper part of the vertical nanowalls and the larger microvoids underneath could contribute to both entrapping air and helping GNW-ZNS suspend liquids, enabling liquid droplets to roll off the surface. GNW-ZNR, which had a hierarchical structure, also displayed a “rose petal effect” with high adhesion (Fig. 3g). In contrast with GNW-ZNS, the ZnO nanorods entirely covered the GNWs on both sides, and the graphene material was no longer exposed in GNW-ZNR. Since ZnO is less hydrophobic than graphene, the CAs of GNW-ZNR were lower, although there were nano-air pockets present in the material. Our results were in good agreement with previous studies, showing that hierarchical and reentrant structures are favorable for obtaining a stable hydrophobic surface with robust liquid-repellent properties^25,27,28^.

**Fig. 4:**
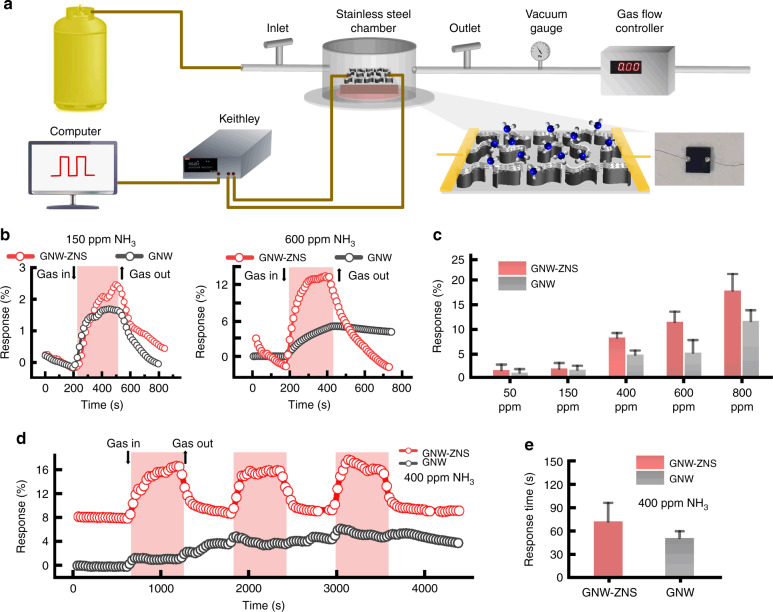
Gas sensing properties of the GNW-ZNS-based sensor. **a** The experimental setup of the gas sensing system. The sensor was placed in a sealed chamber and exposed to premixed gases with different concentrations at a constant flow rate. **b** Representative relative response curves for GNW-ZNS- and GNW-based sensors under NH_3_ at 150 ppm and 600 ppm at RT. **c** Bar graphs of the relative response of GNW-ZNS- and GNW-based sensors to NH_3_ at different concentrations at RT. **d** Dynamic response curves of GNW-ZNS- and GNW-based sensors to NH_3_ (400 ppm) at RT. **e** Quantitative response time of GNW-ZNS- and GNW-based sensors to NH_3_ (400 ppm) at RT

GNW-ZNS consists of graphene and ZnO, and both have been recognized as promising gas-sensitive materials for NH_3_ (refs. ^3,8^). To explore the applicability of the GNW-ZNS structure as a gas sensor, the sample was further integrated into a device with silver wires attached to the surface using silver paste^38^. The device was placed in a sealed chamber (volume: ~0.5 L) and exposed to a series of premixed gases with different concentrations at a constant flow rate of 1000 sccm. The conductivity of the device was recorded at a bias voltage of 1 V using a source meter (Fig. 4a). The relative response (sensitivity) of the gas sensor was evaluated as (*I*_air–_*I*_gas_)/*I*_air_ × 100%, and the recovery rate was evaluated as (*I*_air-after_–*I*_gas_)/(*I*_air_–*I*_gas_) × 100%, where *I*_air_ is the initial conductivity of the sensor in air, *I*_gas_ is the minimum conductivity in the target gas, and *I*_air-after_ is the stable conductivity recovered in air after treatment with the target gas. The response time was defined as the time for the current to achieve a 63.2% change with respect to the minimum conductivity^39^.

**Fig. 5:**
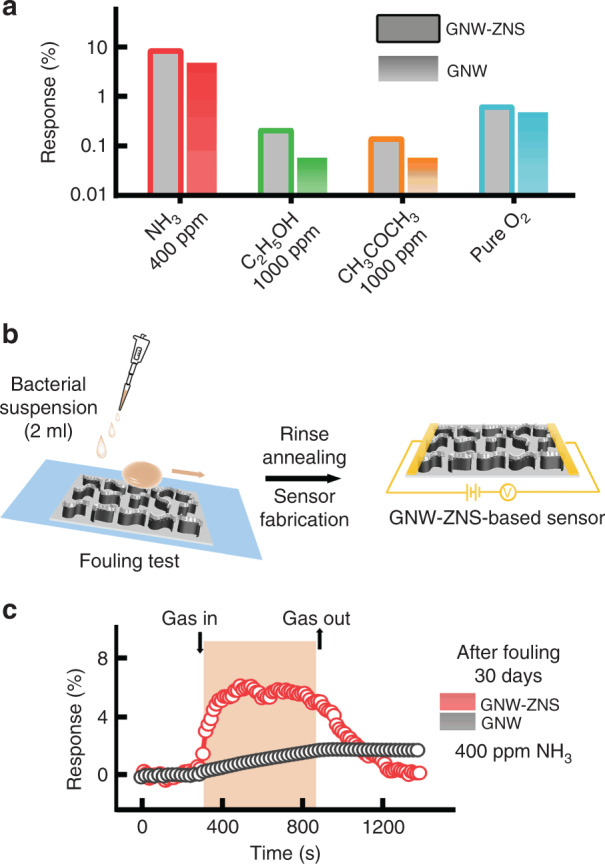
Gas sensing performance against biofouling. **a** Bar graphs of the relative response of GNW-ZNS- and GNW-based sensors to different analytes at RT. **b** Schematic of the biofouling test of GNW-ZNS. Samples were placed on a tilted glass slide, and a bacterial suspension (2 ml) was deposited directly onto the sample surface. The biofouled samples were fabricated into sensors after rinsing and drying, and the gas sensing properties were recorded. **c** Gas response curves of biofouled GNW-ZNS- and GNW-based sensors to NH_3_ (400 ppm) at RT

Figure 4b shows representative relative response curves of GNW-ZNS- and GNW-based devices exposed to NH_3_ at RT. At a relatively low concentration of NH_3_, i.e., 150 ppm, obvious responses were observed for both the GNW-ZNS- and GNW-based devices, with minor differences. The average response was 2.11% for GNW-ZNS and 1.85% for GNW under 150 ppm NH_3_ at RT. The response increased with increasing NH_3_ concentration for both devices. However, different response characteristics could be observed at relatively high NH_3_ concentrations (Fig. 4c). The response increased significantly to ~11.6% for the GNW-ZNS sensor under NH_3_ at 600 ppm, whereas the response of the GNW sensor only increased to 5.3%. Moreover, the recovery rate was significantly decreased for the GNW sensor. As shown in Fig. 4b, the GNW sensor could not recover to its initial level, while good recovery was obtained with the GNW-ZNS sensor. The repeatability of the sensor was also investigated, and the results of the dynamic response for three repeated cycles are shown in Fig. 4d in the case of detecting NH_3_ at a concentration of 400 ppm. The GNW-ZNS sensor displayed good reproducibility with stable recovery. In contrast, the sensing response of the GNW sensor was unstable without a consistent response trend under repeated cycles. The average response times estimated by the dynamic response curves were ~73 s for the GNW-ZNS sensor and 52 s for the GNW sensor under 400 ppm NH_3_. Note that the response time was overestimated due to the limit of our gas inlet configuration in the gas sensing system (Fig. 4e). The above results indicated that compared with the GNW sensor, the GNW-ZNS sensor could respond to NH_3_ over a wider ppm range with better sensitivity and reversibility despite a slightly longer response time. In addition, the selectivity of the sensors was tested against different analytes, including ethanol, acetone, and pure O_2_. Both sensors exhibited relatively low sensitivity toward various interferents, even at high concentrations, demonstrating their good selectivity toward NH_3_ (Fig. 5a).

Furthermore, the excellent liquid repellency of GNW-ZNS may offer the possibility of gas sensing with high stability even under severe conditions. To test this, the gas sensing performance of a biofouled GNW-ZNS sensor was evaluated. A GNW sensor with high liquid adhesion properties was also studied as a control. Fresh GNW-ZNS and GNW samples were placed on a tilted glass slide (10°), and a bacterial suspension was constantly deposited on the surfaces until a total of 2 ml had been deposited. During this treatment, bacterial droplets could slide off the GNW-ZNS sample easily but tended to remain on the GNW sample (Fig. 5b). After rinsing and drying, the samples were further fabricated into sensors, and the gas sensing properties were evaluated. As shown in Fig. 5c, the GNW-ZNS sensor challenged by a bacterial suspension still exhibited a stable response of 6.0% toward 400 ppm NH_3_, a response ~70% that of an untreated GNW-ZNS sensor, which had an average response of 8.4%. In contrast, the GNW sensor after bacterial fouling only showed a response of 1.7%, indicating severe deterioration to only one-third the level of the untreated GNW sensors (4.9% toward 400 ppm NH_3_). In addition, the GNW-ZNS sensor retained excellent recovery, while the GNW sensor could not readily recover its original conductivity. These results demonstrated that the GNW-ZNS sensor can display great stability under severe biofouling conditions, which is challenging to achieve with conventional gas sensors.

The gas sensing mechanism of graphene-based materials has been widely studied, yet it is not fully understood at present. In general, it is agreed that the detection of gas molecules is closely related to the charge transfer from the gas molecules to the sensitive surface, causing a change in the charge-carrier concentration and therefore a change in resistivity^40^. GNW-ZNS consists of graphene and ZnO. Graphene normally exhibits p-type semiconducting behavior, and ZnO is an n-type semiconductor^6^. The gas response characteristics of composites; however, vary in different studies since there are many controlling factors, such as morphology and component contents^15,41–43^. For example, in recent studies, rGO and WO_3_ (n-type) nanocomposites displayed p-type behavior toward NH_3_ detection^41^. Gas sensors fabricated from nanohybrids of rGO and SnS_2_ (n-type) exhibited transitions from p-type to n-type sensing behavior upon adjusting the content of rGO (ref. ^42^).

In this study, both the GNW-ZNS and GNW sensors exhibited p-type behavior^15,44^. It is possible that when GNW-ZNS and GNW were exposed to NH_3_ molecules, the NH_3_ molecules were absorbed on their surfaces, and electrons were transferred from the NH_3_ molecules to the structures due to the strong electron-donating property of the reducing gas NH_3_. Accordingly, the hole carrier concentration in both GNW-ZNS and GNW decreased, leading to reductions in their conductivity. The vertical nanowall structures in GNW-ZNS and GNW possessed a high surface area, allowing NH_3_ molecules to infiltrate the structures through plentiful inter-nanowall channels, thereby providing a good basis for gas detection^7^. The p-type behavior of GNW-ZNS was similar to that of GNW, suggesting that the response properties of GNW-ZNS were dominated by the GNW backbone, probably because the content of ZnO was relatively low. In addition, in GNW-ZNS, ZNS were introduced purposely only at the top of the GNW, and the majority of the graphene was still exposed to contact with NH_3_ molecules, yielding a p-type sensing response.

Compared with the GNW sensor, the GNW-ZNS sensor not only showed a better response over a broader range toward NH_3_, but also better reproducibility and stability. The enhanced gas sensing response could be attributed to the hierarchical and reentrant features of GNW-ZNS. In GNW-ZNS, interfaces between different materials were created after depositing the ZnO layer, and p–n heterojunctions could form between the graphene and ZnO in GNW-ZNS. The electron state in the depletion layers of heterojunctions will change when NH_3_ molecules are in contact with the interfaces, and the charge transfer in GNW-ZNS may be more active than that in GNW (refs. ^15,16^). This behavior may explain why GNW-ZNS can display a good response even at relatively high concentrations of NH_3_. In contrast, the desorption process in the GNW sensor was more difficult. After adsorbing NH_3_ molecules, the sensor had difficulty recovering to its original state. In addition, the numerous ZNS significantly increased the specific surface area and may have resulted in more adsorption sites for NH_3_, which could also be favorable for gas sensing at high concentrations.

Furthermore, the hierarchical and reentrant features of GNW-ZNS provided excellent liquid repellency. When subjected to a severe biofouling challenge by direct deposition of a bacterial suspension, the material could resist contamination more effectively than the other samples and maintain excellent gas sensing ability. Sensors biofouled in relatively static conditions but for a longer time also demonstrated the superior stability of GNW-ZNS than GNW (please see Supplementary Fig. 2 for more details). Overall, acceptable and comparable sensitivity as well as high stability was observed for the GNW-ZNS sensor (Supplementary Table 3), which are desirable characteristics of gas sensors for extended application. It should also be mentioned that at the current stage, the influence of the steric effect of the nanospikes on the gas sensing properties of the GNW-ZNS sensor has not been fully clarified. Further work is required to examine this effect and determine the optimum GNW-ZNS structure in terms of both antifouling and gas sensing properties. Investigating the sensing characteristics of GNW-ZNS sensors with different inter-nanowall distances and comparing the sensing properties of GNW-ZNS with those of GNW-ZF (without hierarchical nanospikes) and GNW-ZNR (without exposed graphene) are possible ways to better study the underlying mechanisms.

## Conclusion

In summary, we have demonstrated the effectiveness of a highly liquid-repellent hierarchical and reentrant graphene-based GNW-ZNS structure for the sensitive detection of NH_3_ at RT. GNW-ZNS features numerous branched ZNS on top of GNWs and can be fabricated by MPECVD, magnetron sputtering and hydrothermal growth. Owing to its unique reentrant features, the hierarchical GNW-ZNS exhibited excellent liquid repellency toward various fluids, such as bacterial suspensions, without surface modification. The GNW-ZNS structure could be further applied as an NH_3_ sensor with reasonable sensitivity over a broad ppm range. The vertical nanowall structure with plentiful inter-nanowall channels offered ample space for gas molecules to infiltrate the structure. The overhanging ZNS increased the surface area, possibly providing more adsorption sites and enhancing the charge transfer efficiency, resulting in a higher sensitivity than that of the GNW sensor. Moreover, the liquid repellency of GNW-ZNS made it possible for the sensor to maintain excellent gas sensing stability even after severe bacterial contamination. The anti-biofouling gas sensors described here provide a facile method to construct reentrant and hierarchical structures with desirable liquid-repellent properties without chemical surface modification. This research also opens new opportunities for designing high-performance gas sensors with resistance to biofouling, which could be applied in complicated bio-environments.

## Materials and methods

The fabrication of GNW-ZNS mainly consisted of three steps. In the first step, GNWs were deposited on Si/SiO_2_ substrates using the MPECVD method. Clean substrates (size: 5 × 5 × 0.5 mm or 10 × 10 × 1 mm) were pretreated with H_2_ and Ar at 800 °C for 15 min. Then, mixed CH_4_ (flow rate: 60 sccm) and H_2_ (flow rate: 10 sccm) were introduced as gas reactants and maintained at 800 °C at a power of 1000 W for 20 min. After cooling to RT under vacuum, GNW samples were obtained. Next, a ZnO layer with a thickness of ~30 nm was prepared via a magnetron sputtering system (ZKDS VTC-300, China) under a vacuum of 2.0 Pa and DC power of 280 W. By magnetron sputtering, the ZnO seed layer was deposited only on the top portion of the GNW. Finally, ZNS were grown on the ZnO seed layer using the hydrothermal growth method in a solution of hexamethylenetetramine (25 mM) and Zn(NO_3_)_2_ (25 mM) at 90 °C for 1.5 h. After the hydrothermal reaction, the samples were rinsed with ethanol and distilled water several times, and annealed in a vacuum oven at 150 °C for at least 24 h. The control sample of GNWs with uniformly distributed ZnO nanorods (denoted as GNW-ZNR) was prepared by ALD and hydrothermal growth. By ALD, a ZnO seed layer with a thickness of ~30 nm was deposited on the GNW sample. Then, ZnO nanorods were grown via a process similar to that used for GNW-ZNS. The GNW-ZNR sample was obtained after rinsing and annealing. Details regarding the fabrication process of GNW and GNW-ZNR can be found in our previous studies^30,31^.

The surface morphology of the obtained structures was characterized by SEM (Zeiss Supra 60). The composition and structure of the samples were investigated by EDS (Oxford Instrument) and Raman spectroscopy (Renishaw inVia Reflex spectrometer system). Wetting properties were studied by measuring the CAs and performing liquid *Escherichia*impacting tests with a goniometer measuring system (Kurss, DSA 15). Static CAs were measured by placing a 4 µl droplet onto the sample surface at RT. Different liquids, including deionized water, blood (whole blood of sheep, RuiTe Company, China), and bacterial suspensions (*Escherichia coli*, ATCC25922, Rui Bio, ~10^7^ colony forming units), were tested as probe liquids. At least three measurements were repeated for each sample to obtain a reliable result. Liquid impacting tests were conducted by recording free-falling liquid droplets (water and bacterial droplets: 5 µl; blood droplet: 12 µl) impacting a tilted sample surface (tilted angle = 2°) from a height of 6 cm.

The gas sensing performance was evaluated using a homemade measurement setup. A sample (substrate size: 5 × 5 mm) was integrated into a device with silver wires attached at two different points on its surface. The contacts to the wires were simply made using conductive silver paste (SCP, No. SCP50G). In a typical gas sensing test, the device was placed in a sealed chamber (volume: ~0.5 L). The electric characteristics of the device were monitored by recording the conductivity continuously at a voltage of 1 V using a source meter (Keithley 2400 or 2601). After a steady current was measured in air, premixed gases were then introduced into the chamber for a certain time at a constant flow rate of 1000 sccm by a gas flow controller (QC-1S, Beijing Municipal Institute of Labour Protection, China). The concentrations of premixed gases, including NH_3_ and ethanol, were calculated according to the injected amount in the gas container and further calibrated using commercial gas sensors (PLT811- NH3, PLT840, Anchuangjia, China).

The relative response (sensitivity) of the gas sensor was evaluated as (*I*_air_–*I*_gas_)/*I*_air_ × 100%, and the recovery rate was evaluated as (*I*_air-after_–*I*_gas_)/(*I*_air_–*I*_gas_) × 100%, where *I*_air_ is the initial conductivity of the sensor in air, *I*_gas_ is the minimum conductivity in the target gas, and *I*_air-after_ is the stable conductivity recovered in air after treatment with the target gas. The response time was defined as the time for the current to achieve a 63.2% change with respect to the minimum conductivity^39^. The reported response was the average value for at least four individual devices.

The stability of the sensors was evaluated by contaminating the samples with a bacterial suspension. Fresh samples were placed on a tilted glass slide (tilted angle = 10°), and *E. coli* bacterial suspensions (~10^7^ colony forming units, 2 ml in total) were deposited directly on the sample surfaces. The samples were then rinsed with distilled water and ethanol three times. After drying, the samples were fabricated into sensors, and the gas sensing properties were then recorded at RT. Additional biofouling tests on a group of GNW-ZNS and GNW sensors were also performed to compare the sensing performance before and after static contamination. Pretested GNW-ZNS and GNW sensors were briefly biofouled, and the sensing properties were tested after bacterial contamination. Bacterial suspension droplets (20 µl) were directly deposited onto the surfaces, and the samples were incubated at 37 °C for 20 min. The bacterial suspension was then removed, and the samples were rinsed and dried. The electrode was reapplied, and the gas sensing performance was retested.

## Supplementary information

SI
