# Supplementary material for: Anti-biofouling NH3 gas sensor based on reentrant thorny ZnO/graphene hybrid nanowalls
Source: Microsyst Nanoeng. 2020 Jul 13;6:41. doi: 10.1038/s41378-020-0151-5 (PMC8433158; doi:10.1038/s41378-020-0151-5)
Supplement: Supplementary file 1 — SI [file 41378_2020_151_MOESM1_ESM.docx]

**Supporting Information for**

**Anti-Biofouling NH_3_ Gas Sensor Based on Re-entrant Thorny ZnO/Graphene Hybrid Nanowalls**

Tian Hang,^1,^† Jiangming Wu,^1,^† Shuai Xiao,^1^ Baohong Li,^1^ Hongbo Li,^1^ Chengduan Yang,^1^ Cheng Yang,^1^ Ning Hu,^1^ Yonghang Xu,^2^ Yu Zhang,^1,*^ Xi Xie^1,3,*^

^1^State Key Laboratory of Optoelectronic Materials and Technologies, School of Electronics and Information Technology, Guangdong Province Key Laboratory of Display Material and Technology, Sun Yat-Sen University, Guangzhou 510006, China

^2^ School of Materials Science and Energy Engineering, Foshan University, Foshan 528000, China.

^3^ The First Affiliated Hospital of Sun Yat-Sen University, Guangzhou 510080, China.

†These authors contributed equally to this work.

*To whom correspondence may be addressed. Corresponding to: Xi Xie, [xiexi27@mail.sysu.edu.cn](mailto:xiexi27@mail.sysu.edu.cn); Yu Zhang, [stszhyu@mail.sysu.edu.cn](mailto:stszhyu@mail.sysu.edu.cn).


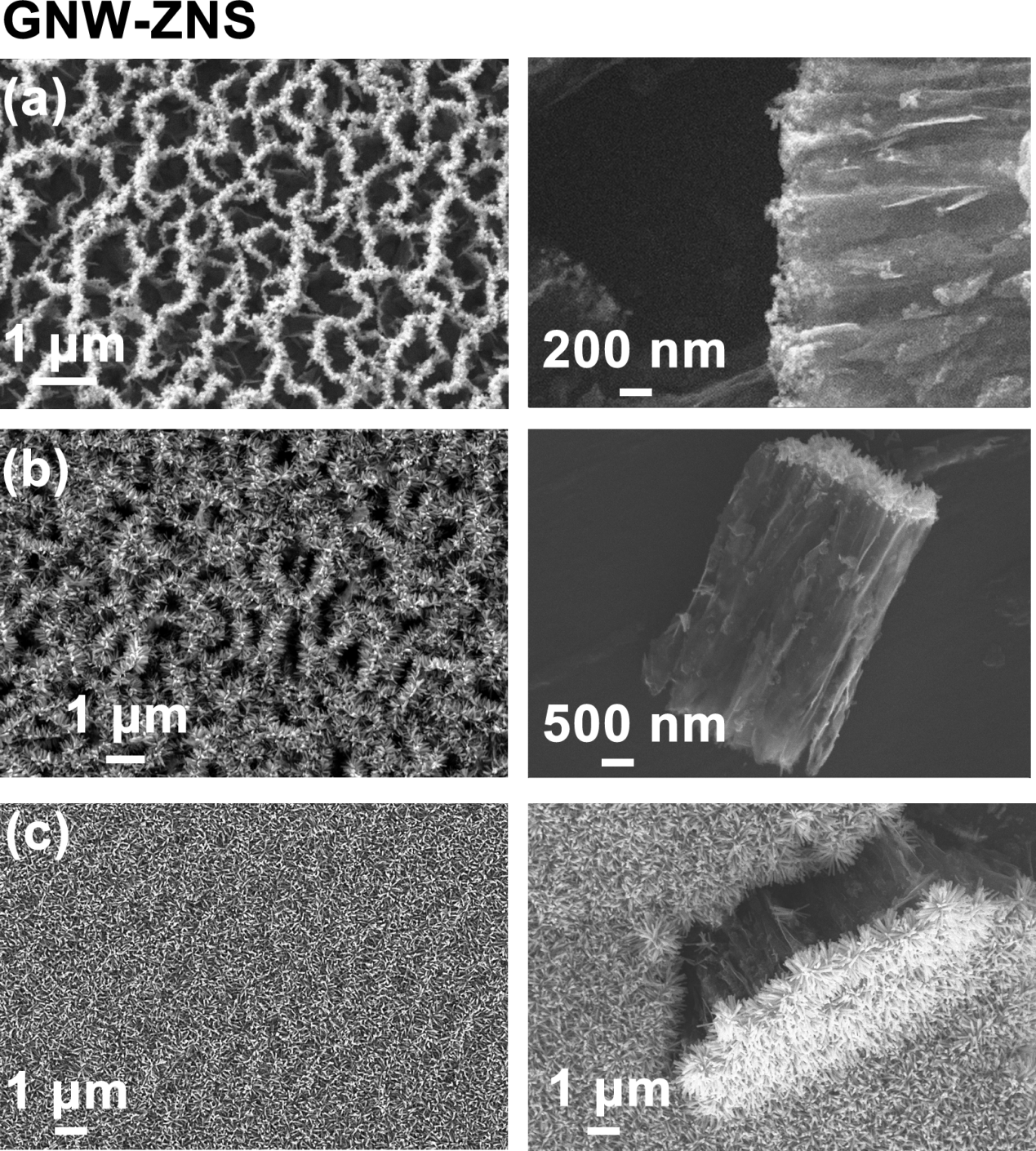


**Figure S1** SEM images of GNW-ZNS with varied inter-nanowall spacings obtained using different hydrothermal growth conditions: (a) in hexamethylenetetramine (HMTA) (10 mM) and Zn(NO_3_)_2_ (10 mM) at 90 °C for 0.5 h; (b) in HMTA (25 mM) and Zn(NO_3_)_2_ (25 mM) at 90 °C for 1.5 h; (c) in HMTA (37.5 mM) and Zn(NO_3_)_2_ (37.5 mM) at 90 °C for 2 h.

|  | **Hydrothermal Growth Condition** | **Average Pore Size**  **(Intersheet distance)**  **(nm)** | **Length of ZnO nanospikes**  **(nm)** | **Diameter of ZnO nanospike (nm)** |
| --- | --- | --- | --- | --- |
| a | HMTA, 10 mM  Zn(NO_3_)_2_, 10 mM  90 °C, 0.5 h | ~ 880 | ~110 | ~40 |
| b | HMTA, 25 mM  Zn(NO_3_)_2_, 25 mM  90 °C, 1.5 h | ~ 780 | ~220 | ~60 |
| c | HMTA, 37.5 mM  Zn(NO_3_)_2_, 37.5 mM  90 °C, 2 h | ~320 | ~670 | ~85 |

**Table S1** Microstructure characteristics of GNW-ZNS tuned using different hydrothermal growth conditions

|  | **SCA/SD (º)** | | |
| --- | --- | --- | --- |
|  | **Distilled Water** | **Blood** | **Bacterial Suspension** |
| **Surface Tension (mN/m)** | 72.3 | 51.6 | 66.3 |
| **GNW-ZNS** | 150.4/6.6 | 139.9/5.9 | 143.6/2.0 |
| **GNW** | 142.6/3.9 | 135.3/7.4 | 141.2/3.6 |
| **GNW-ZF** | 130.9/3.0 | 114.7/3.8 | 127.3/7.9 |
| **GNW-ZNR** | 125.2/13.3 | 127.2/11.8 | 126.9/10.4 |

**Table S2** Results of static contact angle (SCA) measurements


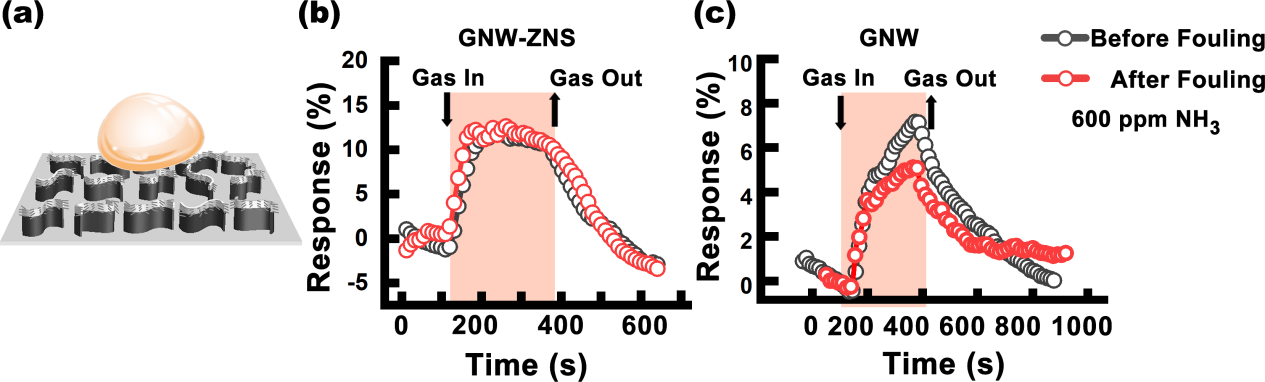


**Figure S2** In order to compare the sensing performance before and after static contamination by bacterial suspension on the same sensors, a group of GNW-ZNS and GNW sensors were bio-fouled shortly and the sensing properties were tested before and after bacterial contamination, respectively. (a) For both sensors, small droplets (20 µl) of bacterial suspension were directly deposited on their surfaces. Different from the contamination with dynamic droplet, the sensors were statically incubated with the droplet at 37 °C for 20 min. After removing the bacterial suspension, rinsing and drying the sensors, the gas sensing performance was re-tested. (b) The GNW-ZNS sensor challenged by a bacterial suspension still exhibited a stable and consistent response of 9.8% towards NH_3_, which was a decrease of less than 10% from the initial response of 10.5%. In contrast, the performance of the GNW sensor severely deteriorated from 7.2% in the initial state to 5.2% after bacterial fouling. In addition, the GNW-ZNS sensor could retain excellent recovery, while the recovery rate of the GNW sensor greatly decreased. The results again demonstrated that the GNW-ZNS sensor possessed better stability under biofouling conditions than the GNW sensor.

| Sensor | NH_3_ Conc. (ppm) | Sensitivity | Response  Time | Ref. |
| --- | --- | --- | --- | --- |
| Reduced graphene oxide/ SnO_2_  nanocrystals | 10000 | 1.46 (I_g_/I_a_) | 30 | [1] |
| ZnO/rGO bilayer thin film | 10 | 1.20 % | 78 | [2] |
| SnO_2_/rGO hybrids | 300 | 4.73% | 100–120 | [3] |
| Graphene Foam | 1000 | 30% | 500 | [4] |
| ZnO–GrO nanocomposite | 1 | 24% | 360 | [5] |
| GNW-ZNS  GNW | 600  600 | 11.6%  5.3% | 73  52 | Current  work |

**Table S3** Comparison of NH_3_ sensing performance of GNW-ZNS- and GNW-based sensors in current work with previous reports

**References:**

1 Mao, S. *et al.* Tuning gas-sensing properties of reduced graphene oxide using tin oxide nanocrystals. *Journal of Materials Chemistry* **22**, 11009-11013, (2012).

2 Tai, H. *et al.* ZnO Nanoparticles/Reduced Graphene Oxide Bilayer Thin Films for Improved NH3-Sensing Performances at Room Temperature. *Nanoscale Research Letters* **11**, 130, (2016).

3 Zhang, D. Z., Liu, J. J., Jiang, C. X., Liu, A. M. & Xia, B. K. Quantitative detection of formaldehyde and ammonia gas via metal oxide-modified graphene-based sensor array combining with neural network model. *Sensors and Actuators B-Chemical* **240**, 55-65, (2017).

4 Yavari, F. *et al.* High Sensitivity Gas Detection Using a Macroscopic Three-Dimensional Graphene Foam Network. *Scientific Reports* **1**, 166, (2011).

5 Singh, G. *et al.* ZnO decorated luminescent graphene as a potential gas sensor at room temperature. *Carbon* **50**, 385-394, (2012).
